# Candidemia in critically ill COVID-19 patients: Risk factors and impact on mortality

**DOI:** 10.1016/j.heliyon.2024.e28033

**Published:** 2024-03-15

**Authors:** Sumeyye Kazancioglu, Hurrem Bodur, Ipek Mumcuoglu, Aliye Bastug, Bahadir Orkun Ozbay, Omer Aydos, Bedia Dinc

**Affiliations:** a Health Science University, Ankara Bilkent City Hospital, Department of Infectious Diseases and Clinical Microbiology, Ankara, Turkey; b Ministry of Health Ankara Bilkent City Hospital, Department of Infectious Diseases and Clinical Microbiology, Ankara, Turkey; c Ministry of Health Ankara Bilkent City Hospital, Medical Microbiology Department, Ankara, Turkey

**Keywords:** Candidemia, COVID-19, Mortality, Intensive care unit, Bloodstream infection

## Abstract

**Background:**

Risk factors of candidemia are well-described in intensive care units (ICUs) before the Coronavirus disease 2019 (COVID-19) pandemic. The increased rates of admission to ICUs have appeared during the pandemic.

**Methods:**

Patient characteristics and laboratory data of 80 candidemia with COVID-19, 101 candidemia without COVID-19, and 100 non-candidemia with COVID-19 patients were evaluated, in this study.

**Results:**

Systemic inflammatory response syndrome (SIRS) ≥ 2, solid malignancy, total parenteral nutrition (TPN), central venous catheterization (CVC), hypotension, fever, urea, alanine aminotransferase (ALT), D-dimer, procalcitonin, ferritin, and delta neutrophil index (DNI) was found to be associated with candidemia in COVID-19 patients. TPN, hypotension, and fever were identified as independent predictors of candidemia in COVID-19, and candidemia in COVID-19 is characterized by significantly high mortality rates. Urea, lactate, and procalcitonin were defined as independent predictors of hospital mortality in candidemia patients with COVID-19.

**Conclusion:**

The presence of candidemia increases mortality in COVID-19. TPN, fever, and hypotension werefound to be the most powerful predictors of candidemia in COVID-19. Overall, these data show that candidemia in COVID-19 is characterized by significantly high mortality rates. Determination of distinctive features can prevent candidemia and mortality.

## Highlights

• TPN, fever, and hypotension are powerful predictors of candidemia in COVID-19.
• The presence of candidemia increases mortality in COVID-19.
• Urea, lactate, and, procalcitonin were defined as independent predictors of mortality in candidemia patients with COVID-19.

## 1 Introduction

Invasive fungal infections are increasing due to more immunosuppressive treatment and invasive procedures. Candidemia is a blood-stream infection, seen at increasing rates in intensive care units (ICUs) which causes high mortality [[1], [2], [3]]. The risk factors for Candida infection have been established in several studies. The most significant risk factors are critical illness, surgery, pancreatitis, malignancies, solid organ transplantation, the use of broad-spectrum antibiotics (like carbapenems), central venous catheterization, total parenteral nutrition, hemodialysis, and ımmunosupressive agents [1,[4], [5], [6]].

With the emergence of the Coronavirus disease 2019 (COVID-19) pandemic, increased rates of admission to ICUs have appeared [7]. Increased candidemia incidence was previously reported in COVID-19 and non-COVID-19 patients during the pandemic [[8], [9], [10]]. Therefore, knowledge of patients' characteristics and risk factors associated with candidemia in COVID-19 is limited. Many factors can be associated with candidemia in critically ill COVID-19 patients. COVID-19 patients in ICUs have higher mortality rates and fungal pathogen coexistence with COVID-19 leads to an increase in the high mortality rates [7,11,12]. Identification of patients at risk for candidemia is crucial to minimize the mortality in COVID-19.

First, this study aimed to identify candidemia risk factors in patients with COVID-19 in ICUs. Second, we analyzed hospital mortality for candidemia patients with COVID-19 and evaluated them with non-COVID-19 controls.

## 2 Methods

### 2.1 Study design and participants

The present study retrospectively enrolled 80 candidemia patients with COVID-19 who were hospitalized in the ICUs of Ankara City Hospital from April 01, 2020 to March 01, 2021. The case (Candidemia with COVID-19) group included ICU patients with the diagnosis of COVID-19 pneumonia if they were ≥16 years old with at least one blood culture positive for *Candida* spp.. The control (Non-candidemia COVID-19) group (n = 100) included patients with COVID-19 pneumonia (≥16 years) during the same period and the same ICUs with negative blood cultures for candidemia. The diagnosis of COVID-19 was confirmed by detecting severe acute respiratory syndrome coronavirus-2 (SARS-CoV-2) RNA in oro-nasopharyngeal swab samples by PCR. All COVID-19 patients had PCR confirmation and radiologic findings of thorax computed tomography (CT) investigation. Also, 101 candidemia episodes in patients who hospitalized with another diagnosis except for COVID-19 (After emerging of COVID-19, All patients included in the study tested negative for SARS-CoV-2 PCR and had no pathological lung findings compatible with COVID-19) from April 1, 2019 to March 1, 2021 were included as Candidemia without COVID-19 group.

An episode of candidemia was defined as the isolation of *Candida* spp. from a blood culture and, episodes of recurrent candidemia were excluded. Results of positive blood cultures for candidemia were extracted from the microbiology laboratory.

### 2.2 Laboratory tests

Blood cultures are loaded into a BacT/ALERT 3D Microbial Detection System (BioMerieux, Inc., NC). The Vitek2 system was used for yeast identification according to the manufacturer's instructions. The Vitek MS was used without prior protein extraction. A portion of one colony isolated from a Sabouraud agar plate (bioMérieux, Marcy l'Etoile, France) was applied directly onto the Vitek MS disposable target (single deposit) and was lysed with 0.5 μl of 25% formic acid. After drying completely at room temperature (1–2 min), 1 μl of ready-to-use α-cyano-4-hydroxycinnamic acid (CHCA) matrix (bioMérieux, Marcy l'Etoile) was applied to the spot, which was allowed to dry completely again (1 min).

### 2.3 Data collection and definitions

Demographic data and laboratory values were extracted from electronic medical records and patients’ files. The following variables were recorded for each patient: age, gender, Charlson comorbidity index (CCI) and systemic inflammatory response syndrome (SIRS) score, presence of various known risk factors for candidiasis (for example, an indwelling central venous catheterization (CVC), antibiotic use, malignancy, steroid administration, mechanical ventilation (MV), hemodialysis, hospitalization time in ICU, total parenteral nutrition (TPN), surgery, candiduria), fever and hypotensive conditions, hospital mortality. Laboratory results of the patients on admission were recorded. Laboratory values (a complete blood count (CBC), C-reactive protein (CRP), procalcitonin, alanine aminotransferase (ALT), aspartate aminotransferase (AST), urea, creatinine, creatinine kinase (CK), lactate dehydrogenase (LDH), international normalization ratio (INR), activated partial thromboplastin time (aPTT), D-dimer, lactate, ferritin were recorded.

### 2.4 Statistical analysis

Continuous data were presented as median (minimum-maximum) and categorical data were presented as percentages. Statistical analyses were performed using the SPSS software version 24.0 (IBM Corporation, Armonk, NY, USA). Comparisons for categorical variables were executed using the Pearson chi-square test or Fisher's exact test. The Kolmogorov-Smirnov test was performed to check the normality of the continuous variables. Differences between the two groups were compared using the Mann-Whitney *U* test. Kruskal Wallis test was used for comparisons of more than two groups and the significant (*P* < 0.05) results from the Mann-Whitney test (with Post hoc Bonferroni correction) were performed. Factors associated with candidemia in COVID-19 patients were analyzed by using univariate and multivariate logistic regression (The Forward LR (Likelihood Ratio)) analysis. When selecting the parameters to be included in multivariate analysis, the *P*-value cut-off was determined as 0.05. Taking into account the parameters reported to have an effect on mortality in COVID-19 and candidemia previously, survivors and non-survivors groups were compared for all three groups. Univariate logistic regression analysis was performed for mortality in candidemia patients with COVID-19. A Cox regression was used to determine independent predictors for mortality in candidemia patients with COVID-19. Parameters found to be statistically significant between survivors and non-survivors groups were entered in the multivariate model (using a cut-off *P*-value of 0.05). Statistical significance was defined as *P* < 0.05.

### 2.5 Ethics statement

Approval of the ethics committee by Ankara City Hospital was obtained for this study (confirmation date and number: February 17, 2021/E1-21-1520). Informed consent was not required for this study because it is a retrospective design and the data of the study consists of routine examinations. This study was conducted by the principles of the Declaration of Helsinki.

## 3 Results

### 3.1 Candida species distribution

In candidemia patients with COVID-19 (n = 80), the distribution of Candida species was as follows: *C. albicans* 46.25% (n = 37), *C. parapsilosis* 21.25% (n = 17), *C. glabrata* 13.75% (n = 11), *C. tropicalis* 12.50% (n = 10), *C. kefyr* 1.25% (n = 1), *C. lusitaeniea* 1.25%(n = 1), *C. krusei* 1.25% (n = 1), *C. dublinensis* 1.25% (n = 1) and, *C. orthopsilosis* 1.25% (n = 1).

In candidemia patients without COVID-19 (n = 101), the distribution of Candida species was as follows: *C. albicans* 41.58% (n = 42), *C. parapsilosis* 23.76% (n = 24), *C. glabrata* 15.84% (n = 16), *C. tropicalis* 11.88% (n = 12), *C. kefyr* 1.98% (n = 2), *C. dublinensis* 1.98% (n = 2), *C. lusitaeniea* 0.99% (n = 1), *C. guilliermondii* 0.99% (n = 1) and, *C. krusei* 0.99% (n = 1).

### 3.2 Clinical characteristics and laboratory parameters of the study population

In total, 80 candidemia with COVID-19, 101 candidemia without COVID-19, and 100 non-candidemia with COVID-19 patients participated in the study. There were no significant differences in median age and sex between the three groups. In candidemia patients with COVID-19, the median time from hospitalization to candidemia was shorter than that of non-COVID-19 candidemia patients. Candidemia patients with COVID-19 had higher percentages of MV support and steroid administration than non-COVID-19 candidemia patients. Between candidemia groups; the COVID-19 candidemia group had higher CK, AST, ALT, LDH, delta neutrophil index (DNI), and neutrophil-to-lymphocyte ratio (NLR) than the non-COVID-19 candidemia group.

In the COVID-19 groups, according to the presence of concurrent candidemia; the percentages of SIRS ≥2, solid malignancy, surgery, steroid administration, MV, TPN, CVC, hypotension, and fever were higher in the COVID-19 candidemia group than non-candidemia COVID-19 group, significantly. There were several significant differences in laboratory parameters between COVID-19 groups; including lower platelets and higher urea, CK, ALT, INR, aPTT, D-dimer, procalcitonin, ferritin, leucocytes, DNI was found in the candidemia patients than non-candidemia patients with COVID-19 (Table 1, Table 2).

**Table 1:** Characteristics of patients with and without candidemia.

	Candidemia Non-COVID-19 (n = 101)		Candidemia COVID-19 (n = 80)		Non-candidemia COVID-19 (n = 100)		X^2^	*P* -value
Age, years	69	(23–93)	70.5	(20–92)	68	(33–97)	0.609	0.737
Male gender	57	(56.4)	47	(58.8)	53	(53)	0.616	0.735
Clinical Conditions								
Hypotension	86	(85.1)^c^	68	(85)^c^	23	(23)^**a,b**^	106.494	***0.000***
Fever	47	(46.5)^c^	29	(36.3)^c^	7	(7)^**a,b**^	40.157	***0.000***
SIRS^‡^ ≥ 2	98	(97)^c^	79	(98.8)^c^	83	**(83)**^**a,b**^	20.568	***0.000***
Charlson Comorbidity index	5	(0–11)^c^	5	(0–10)	4	(0–11)^a^	9.229	***0.010***
Comorbidities								
Hematologic malignancy	4	(4)	0	(0)	2	(2)	2.991	0.245
Solid malignancy	25	(24.8)^c^	12	(15.2)^c^	4	(4)^**a,b**^	17.340	***0.000***
Diabetes	31	(30.7)	33	(41.8)	41	(41)	3.135	0.209
Chronic renal failure	8	(7.9)	8	(10.1)	8	(8)	0.340	0.844
Hemodiaylis	6	(5.9)	6	(7.6)	5	(5)	0.599	0.775
Neurologic disease	25	(24.8)^c^	14	(17.7)	10	(10)^**a**^	7.578	***0.023***
Coronary arterial disease	30	(29.7)	24	(30.8)	37	(37)	1.385	0.500
Chronic obstructive lung disease	11	(10.9)	12	(15.2)	16	(16)	1.240	0.538
Obesity	1	[1]	1	(1.3)	2	(2)	0.591	0.838
Predisposing Factor								
Total parenteral nutrition	27	(27)^**c**^	22	(27.5)^c^	4	(4)^**a,b**^	22.598	***0.000***
Central venous catheterization	79	(78.2)^c^	68	(85)^c^	60	(60)^a,b^	16.003	***0.000***
Mechanical ventilation	57	(57)^b^	61	(76.3)^a,c^	56	(56)^b^	9.496	***0.009***
Surgery within the past 3 months	35	(34.7)^b,c^	10	(12.7)^a,c^	4	[4]^a,b^	34.490	***0.000***
Antibiotic use	97	(96)	80	(100)^c^	92	(92.9)^b^	6.191	***0.035***
Steroid administration	3	(3)^**b,c**^	62	(78.5)^a,c^	76	(76)^a,b^	84.136	***0.000***
Fluconazole prophylaxis	5	(5)	6	(7.5)	5	(5.1)	0.717	0.763
Candiduria	20	(19.8)	21	(26.3)	12	(24)	1.090	0.580
Length of ICU^†^ stay, days	31	(2–161)^b,c^	20	(2–105)^a,c^	14	(6–47)^a,b^	44180	***0.000***
Time from hospitalization to candidemia, days	22	(2–183)	17	(2–60)	–	–	15.397	***0.000***
Time of death from candidemia, days	6	(1–63)	5	(1–53)	–	–	1.762	0.184
Time of death from hospitalization, days	36.5	(6–240)^b,c^	20.5	(1–76)^a^	19	(9–50)^a^	24.870	***0.000***
Hospital mortality	66	(65.3)^b,c^	64	(80)^a,c^	51	(51)^a,b^	16.366	***0.000***

Pearson Chi-Square, Fisher's Exact test, Kruskal Wallis h analysis. Data are n (%) or median (min-max). ^†^ ICU, ıntansive care unit; ^‡^ SIRS, systemic inflammatory response syndrome. (a) Statistically significant than Candidemia Non-COVID-19 group, (b) Statistically significant than Candidemia COVID-19 group, (c) Statisticallysignificant than Non-Candidemia COVID-19 group.

**Table 2:** Laboratory parameters of patients with and without candidemia.

		Candidemia Non-COVID-19 (n = 101)	Candidemia COVID-19 (n = 80)	Non-candidemia COVID-19 (n = 100)	X^2^	*P* -value
	Normal Range (Unit)	median (min.-max.)	median (min.-max.)	median (min.-max.)		
Urea	20-49 (mg/dL)	62 (12–276)	79 (13–529)^c^	51 (15–244)^b^	13.221	***0.001***
Creatinine	0.7–1.3(mg/dL)	0.97 (0.15–7.3)	0.99 (0.24–8.91)	0.88 (0.37–4.13)	2.931	0.231
Creatinine Kinase	32-294 (U/L)	50.5 (5–22790)^b^	131.5 (27–2600)^a,c^	87 (4–1203)^b^	13.041	***0.001***
Aspartate aminotransferase	<35 (U/L)	36.5 (9–3416)^b^	52.5 (15–3564)^a^	45.5 (14–756)	10.585	***0.005***
Alanine aminotransferase	<50 (U/L)	23 (3–905)^b^	40.5 (4–3185)^a,c^	29 (5–218)^b^	11.706	***0.003***
Lactate dehidrogenase	120-246 (U/L)	346 (136–5061)^b,c^	547 (194–12269)^a^	523.5 (184–1024)^a^	41.360	***0.000***
Total bilirubin	0.2–1.0 (mg/dL)	0.8 (0.1–13.8)^c^	0.7 (0.2–15.3)	0.6 (0.1–3.7)^a^	8.255	***0.016***
International normalized ratio	0.8–1.2	1.35 (0.87–1392)^b,c^	1.19 (0.92–4.21)^a,c^	1.12 (0.82–2.3)^a,b^	41.710	***0.000***
Activated partial thromboplastin time	21-32 sn	30.4 (2.29–120)^c^	29.25 (19.2–120)^c^	24.2 (2–445.9)^a,b^	36.694	***0.000***
D-dimer	<0.55 mg/L	4.6 (1–35.2)^c^	3.97 (0.4–35.2)^c^	1.2 (0.19–35.2)^a,b^	41.962	***0.000***
Procalcitonin	<0.16 μg/l	0.9 (0.07–126)^c^	0.59 (0.03–177)^c^	0.17 (0.02–80)^a,b^	65.580	***0.000***
Ferritin	10–291 μg/L.	1063 (205–10720)	1094 (114–140162)^c^	522.5 (28–6295)^b^	14.382	***0.001***
Leucocytes	4200-10800 (μ/L)	9100 (260–49350)	11635 (3200–38000)^c^	9650 (1990–25750)^b^	6.022	***0.049***
Neutrophils	1700-7900 (μ/L)	7700 (60–46290)	9750 (2600–32000)	8490 (1880–24790)	5.646	0.059
Lymphocytes	1500-4500 (μ/L	760 (130–2940)^c^	665 (90–3820)	560 (40–7900)^a^	13.366	***0.001***
Monocytes	100-900 (μ/L)	360 (30–2680)	415 (60–1400)^c^	310 (30–1110)^b^	8.258	***0.016***
Platelets	16000-383000 (μ/L)	211 (10–805)^c^	195.5 (23–571)^c^	270 (81–637)^a,b^	21.886	***0.000***
DNI^†^	%	1.2 (0.1–55.5)^b^	2.3 (0.1–22.3)^a,c^	0.9 (0.1–11.4)^b^	16.388	***0.000***
Lactate	0.5–1.0 mmol/L	1.68 (0.36–12.37)	1.88 (0.55–11.77)	2.07 (0.12–32.7)	3.453	0.178
C-reactive protein	0-5 (g/L)	119.5 (3–305)	97.5 (1–415)	121 (1–344)	3.336	0.189
Neutrophil-to-lymphocyte ratio	–	10.55 (0.35–69.09)^b,c^	14.72 (1.44–110.93)^a^	13.6 (0.72–125)^a^	8.769	***0.012***
Lymphocyte-to-C-reactive protein ratio	–	7.02 (0.93–576.67)^c^	7.3 (0.6–470)	4.9 (0.46–240)^a^	8.733	***0.013***

Kruskal Wallis h analysis. ^†^ DNI, Delta neutrophil index. Data are n (%) or median (min-max). (a) Statistically significant than Candidemia Non-COVID-19 group, (b) Statistically significant than Candidemia COVID-19 group, (c) Statistically significant than Non-Candidemia COVID-19 group.

### 3.3 Risk factors for candidemia in COVID-19 patients

Univariate logistic regression analysis was performed for the predictors of candidemia in COVID-19 patients. All parameters that were statistically significant between COVID-19 groups were taken into the analysis. SIRS ≥2, solid malignancy, TPN, CVC, hypotension, fever, urea, ALT, D-dimer, procalcitonin, ferritin, and DNI were found to be associated with candidemia in COVID-19. Multivariate logistic regression analysis showed that TPN, hypotension, and fever were identified as independent predictors of candidemia in COVID-19 patients (Table 3).

**Table 3:** Risk factors for candidemia in COVID-19 patients.

	Univariate					Multivariate				
	B	*P* -value	OR	95% CI		B	*P* -value	OR	95% CI	
SIRS^a^ ≥ 2	2.784	***0.007***	16.181	2.104	124.462					
Solid malignancy	1.458	***0.015***	4.299	1.329	13.903					
Total parenteral nutrition	2.209	***0.000***	9.103	2.988	27.737	2.878	***0.006***	17.778	2.323	136.054
Central venous catheterization	1.329	***0.000***	3.778	1.816	7.859					
Hypotension	2.943	***0.000***	18.971	8.780	40.990	3.306	***0.000***	27.285	5.980	124.489
Fever	2.022	***0.000***	7.555	3.092	18.456	2.608	***0.004***	13.576	2.270	81.185
Urea	0.011	***0.000***	1.011	1.006	1.017					
Alanine aminotranferase	0.013	***0.006***	1.013	1.004	1.022					
D-dimer	0.077	***0.002***	1.080	1.029	1.133					
Procalcitonin	0.069	***0.035***	1.072	1.005	1.143					
Ferritin	0.0005	***0.016***	1.000	1.000	1.001					
Delta neutrophil index	0.256	***0.000***	1.291	1.138	1.466					

a SIRS, systemic inflammatory response syndrome. OR; Odds ratio. CI; Confidence Interval.

Although the steroid administration no was found to be significantly different between the COVID-19 groups (Candidemia with COVID-19 n = 62 (78.5%) vs. Non-candidemia COVID-19 n = 76 (76%), *P* = 0.01), there was no association found between steroid administration and candidemia in COVID-19 ([OR]: 0.87; 95% CI: 0.43–1.76, p = 0.695).

### 3.4 Clinical characteristics and laboratory parameters for mortality of the study population

The percentage of hospital mortality was higher in candidemia patients with COVID-19 than other two groups (Table 1). In candidemia patients with COVID-19; MV, hypotension, and higher procalcitonin, lactate, CRP, NLR, urea, and lower lymphocyte-to-C-reactive protein ratio (LCR) were found in the non-survivors than survivors. In candidemia patients without COVID-19; MV, hypotension, CCI, SIRS ≥2, and higher aPTT, procalcitonin, CRP, and lower LCR were found in the non-survivors than survivors. In COVID-19 patients without candidemia; age, SIRS ≥2, MV, hypotension, CVC, CCI, higher aPTT, and lower platelets were found in non-survivors than survivors (Table 4).

**Table 4:** Characteristics and laboratory parameters of patients with and without candidemia according to mortality.

	Candidemia Non-COVID-19 (n = 101)			Candidemia COVID-19 (n = 80)			Non-candidemia COVID-19 (n = 100)		
	Survivors	Non-survivors	*P* -value	Survivors	Non-survivors	*P* -value	Survivors	Non-survivors	*P* -value
	(n = 35)	(n = 66)		(n = 16)	(n = 64)		(n = 49)	(n = 51)	
Age (≥65)	20 (57.1)	44 (66.7)	0.344	10 (62.5)	46 (71.9)	0.545	25 (51)	38 (74.5)	***0.015***
Age, years	68 (23–89)	69.5 (27–93)	0.215	66.5 (20–90)	72 (42–92)	0.094	66 (33–97)	71 (36–94)	***0.006***
Male gender	23 (65.7)	34 (51.5)	0.171	8 (50)	39 (60.9)	0.427	23 (46.9)	30 (58.8)	0.234
SIRS^†^ ≥ 2	32 (91.4)	66 (100)	***0.039***	15 (93.8)	64 (100)	0.200	36 (73.5)	47 (92.2)	***0.013***
Mechanical ventilation	14 (41.2)	43 (65.2)	***0.022***	8 (50)	53 (82.8)	***0.017***	7 (14.3)	49 (96.1)	***0.000***
Central venous catheterization	25 (71.4)	54 (81.8)	0.229	14 (87.5)	54 (84.4)	1.000	22 (44.9)	38 (74.5)	***0.003***
Hypotension	24 (68.6)	62 (93.9)	***0.001***	9 (56.3)	59 (92.2)	***0.002***	1 [2]	22 (43.1)	***0.000***
Length of ICU^‡^ stay, days	40 (3–161)	27.5 (1–105)	***0.035***	39.5 (0–105)	18 (1–60)	***0.008***	12 (7–47)	16 (6–45)	***0.008***
Charlson comorbidity index	4 (0–14)	3 (0–7)	***0.033***	3.5 (0–5)	2 (0–9)	0.580	3 (0–7)	5 [[1], [2], [3], [4], [5], [6], [7], [8], [9], [10], [11]]	***0,003***
Time from hospitalization to candidemia, days	4 [[2], [3], [4], [5], [6], [7], [8], [9], [10], [11]]	6 [[2], [3], [4], [5], [6], [7], [8], [9], [10], [11]]	***0.022***	4 [[2], [3], [4], [5], [6], [7], [8], [9], [10]]	5 [[2], [3], [4], [5], [6], [7], [8], [9]]	0.154	–	–	–
Urea	56.5 (21–195)	76.5 (12–276)	0.198	56 (13–238)	97.5 (15–529)	***0.017***	51 (21–150)	54 (15–244)	0.133
aPTT^§^	27.9 (2.29–44.5)	31.1 (21–120)	***0.012***	24.4 (19.2–120)	30 (20–89.8)	0.052	22.9 (2–81.8)	25.5 (19.1–445.9)	***0.001***
Procalcitonin	0.38 (0.07–126)	1.09 (0.07–23.9)	***0.029***	0.23 (0.03–10.56)	1.17 (0.03–177)	***0.006***	0.15 (0.03–80)	0.18 (0.02–2.01)	0.440
Lactate	1.76 (0.85–10.9)	1.66 (0.36–12.37)	0.552	1.45 (0.82–2.1)	2.39 (0.55–11.77)	***0.000***	2.1 (0.84–32.7)	1.92 (0.12–8.21)	0.203
C-reactive protein	103 (3–305)	129 (28–302)	***0.029***	57.5 (1–253)	105.5 (10–415)	***0.049***	122 (1–344)	118 (11–283)	0.845
NLR^¶^	9.74 (0.85–30.46)	11.83 (0.35–69.09)	0.157	8.96 (2.5–77.27)	15.7 (1.44–110.93)	***0.033***	13.18 (0.72–83.76)	14.92 (3.95–125)	0.735
LCR^¶¶^	9.92 (1.67–576.67)	5.93 (0.93–96.43)	***0.015***	11.31 (1.02–470)	6.3 (0.6–105.88)	***0.032***	5.34 (0.88–240)	4.75 (0.46–30)	0.320

Pearson Chi-Square, Fisher's Exact test, Mann Whitney U analysis. Data are n (%) or median (min-max). ^†^ SIRS, systemic inflammatory response syndrome; ^‡^ ICU, intensive care unit; ^§^ aPTT, activated partial thromboplastin time; ^¶^ NLR, Neutrophil-to-lymphocyte ratio; ^¶¶^ LCR, Lymphocyte-to-C-reactive protein ratio.

### 3.5 Risk factors for mortality with candidemia in COVID-19 patients

Univariate logistic regression analysis was performed for the predictors of mortality in all groups. Gender was not associated with mortality in all three groups.

In candidemia patients without COVID-19; MV, hypotension, the day of antifungal treatment from candidemia, and CCI were significantly associated with mortality.

In COVID-19 patients without candidemia; age, SIRS ≥2, MV, hypotension, CCI, and, CVC, were significantly associated with mortality.

In candidemia patients with COVID-19; MV, and, hypotension were found to be significantly associated with mortality. All parameters that were statistically significant between survivors and non-survivors in candidemia patients with COVID-19 were taken into the Cox regression analysis for mortality. Urea, lactate, and procalcitonin levels were defined as independent predictors of mortality in candidemia patients with COVID-19 (Table 5, Table 6).

**Table 5:** Risk factors for mortality of patients.

	Candidemia Non-COVID-19					Candidemia COVID-19					Non-candidemia COVID-19				
	B	*P* -value	Exp(B)	95% CI		B	*P* -value	Exp(B)	95% CI		B	*P* -value	Exp(B)	95% CI	
Age	0.203	0.346	1.225	0.804	1.867	0.214	0.466	1.238	0.697	2.200	0.516	***0.016***	1.675	1.099	2.553
Gender	−0.295	0.173	0.745	0.487	1.138	0.222	0.429	1.249	0.720	2.166	0.240	0.235	1.271	0.856	1.887
SIRS^a^ ≥ 2	10.963	0.999	57722.723	0.000		11.327	1.000	83022.238	0.000		0.723	***0.018***	2.060	1.129	3.757
Mechanical ventilation	1.572	***0.009***	4.818	1.487	15.612	1.572	***0.009***	4.818	1.487	15.612	4.990	***0.000***	147.000	28.957	746.239
Day of treatment for candidemia	−0.275	***0.017***	0.760	0.606	0.953	−0.204	0.206	0.816	0.595	1.119	–	***-***	–	–	–
Charlson comorbidity index	0.165	***0.025***	1.179	1.021	1.362	0.040	0.763	1.041	0.804	1.347	0.344	***0.003***	1.411	1.124	1.771
Central venous catheterization	0.588	0.232	1.800	0.687	4.719	−0.260	0.755	0.771	0.151	3.929	1.277	***0.003***	3.587	1.542	8.349
Hypotension	1.961	***0.002***	7.104	2.061	24.490	2.217	***0.001***	9.178	2.391	35.226	3.595	***0.001***	36.414	4.658	284.660

a SIRS, systemic inflammatory response syndrome.

**Table 6:** Cox regression analysis for mortality in candidemia patients with COVID-19.

	B	SE	Wald	df	*P* - value	Exp(B)	95 % CI	
Mechanical ventilation	0.426	0.333	1.634	1	0.201	1.531	0.797	2.941
Hypotension	0.498	0.470	1.123	1	0.289	1.646	0.655	4.138
Lactate	0.997	0.334	8.925	1	***0*.*003***	2.711	1.409	5.217
Urea	1.143	0.472	5.866	1	***0*.*015***	3.136	1.244	7.908
Procalcitonin	0.741	0.315	5.531	1	***0*.*019***	2.097	1.131	3.888
C-reactive protein	0.612	0.402	2.320	1	0.128	1.845	0.839	4.057
LCR a	0.728	0.430	2.871	1	0.090	2.071	0.892	4.805
NLR¶	0.004	0,007	0,385	1	0,535	1004	0,990	1019

¶ NLR, Neutrophil-to-lymphocyte ratio.

a LCR, Lymphocyte-to-C-reactive protein ratio.

## 4 Discussion

This retrospective case-control study was designed to analyze risk factors for candidemia in COVID-19. Also, the parameters were investigated for mortality in COVID-19 patients with candidemia. The study represents TPN, hypotension, and fever were the most powerful predictors for candidemia in COVID-19 patients. Urea, lactate, and procalcitonin levels were found to an independent predictors of hospital mortality in candidemia patients with COVID-19.

In this study, non-albicans Candida species was detected at a higher percentege in patients with and without COVID-19.

In a meta-analysis of studies from European countries, it was reported that *C. albicans, C. glabrata, and, C. parapsilosis* were the three species which is similar to the results of the present study [18]. Consistent with this study, Routsi et al. reported that the proportion of non-albicans Candida species was higher in both COVID-19 and pre-COVID-19 cohorts. However, they did not show a difference in species distribution between the pre-pandemic period and the COVID-19 period [19].

Many critically ill COVID-19 patients have common risk factors predisposing them to candidemia which had been previously described [20]. TPN has been identified as the most well-described risk factor for candidemia in numerous studies before the pandemic [1,5,6,15]. Similar to these findings TPN was defined as an independent predictor for candidemia in COVID-19 patients, in the current study. Fever and hypotension take part in severe sepsis and septic shock definitions [21]. It was previously reported that the presence of fever, sepsis, and/or septic shock was associated with candidemia [1,6,22]. In this study, SIRS ≥2 and procalcitonin were found to be associated with candidemia in COVID-19 patients. These findings suggest that critically ill COVID-19 patients who have clinical signs and laboratory findings compatible with sepsis were at greater risk for candidemia. It was reported that critically ill COVID-19 patients whose fever continues despite antibiotic treatment, tests can be planned for fungal agents and antifungal prophylaxis can be given [12,23].

Other parameters that were found to be associated with candidemia in COVID-19 patients were; solid malignancy, CVC, urea, ALT, D-dimer, ferritin, and, DNI. Clinicians can use these parameters to predict candidemia in patients with COVID-19. White et al. showed no relation between underlying co-morbidities and yeast infection in COVID-19(12). In this study, the highest percentage of solid malignancy was in the non-COVID-19 candidemia group. Additionally, solid malignancy was related to candidemia in COVID-19 (OR: 4.3, *P* = 0.015) in this study. A previous study which included only 21 COVID-19 patients with candidemia, showed that the percentage of solid malignancy (45.1%) was significantly higher in the non-COVID-19 candidemia patients than in the COVID-19 candidemia patients (0%) [24]. Macauley et al. showed a higher median day of CVC dwell in candidemia patients with COVID-19 than in non-COVID-19 controls, but they did not evaluate the relation between candidemia and CVC [25]. Previously, the presence of a central venous catheterization (CVC) was defined as an independent risk factor for candidemia in COVID-19 patients [13]. In this study CVC was not found to be an independent risk factor, however, CVC and candidemia were found to be related (OR: 3.8, *P* < 0.001).

The DNI (a hemogram parameter routinely measured in our center laboratory), which is a calculated parameter that reflects the ratio of immature granulocytes over total neutrophil count in the blood, is significant in predicting mortality for sepsis in various studies [17]. Also, it was previously reported to be more predictive of candidemia than leukocytes, CRP, and procalcitonin [26]. In this study, higher medians of DNI were observed in both candidemia groups (with COVID-19 and without COVID-19) than in COVID-19 non-candidemia controls. The DNI may be a useful parameter to predict candidemia in COVID-19 (OR:1.3, *P* < 0.001).

A systematic review revealed that hospital mortality of COVID-19 patients in ICUs was 41% [27]. According to the current study, the coexistence of candidemia with COVID-19 results in higher hospital mortality rates (80%) than candidemia without COVID-19 (65.3%) and COVID-19 without candidemia (%51) groups. It is important and necessary predict to candidemia so as not to increase the current high mortality rates. Similar to the current study, hypotension was previously defined as a risk factor for mortality in candidemia patients [[16], [28], [29]]. Candidemia patients with COVID-19 have a higher percentage of MV support than control groups (76% vs. 57% and 76% vs. 56%, *P* = 0.009, respectively). Nucci et al. found that COVID-19 patients with candidemia were more under MV than non-COVID-19 controls (100% vs. 34.4%, *P* < 0.001) [8]. Bishburg et al. reported that they found a prolonged MV time in eight candidemia patients with COVID-19 than controls [30]. Charlson comorbidity index was found to be associated with mortality in non-COVID-19 candidemia patients (OR: 1.18, *P* = 0.025) in this study. Asai et al. showed a relation between CCI ≥3 and mortality in candidemia patients [31]. In a recent study that analyzed 189 candidemia episodes; the CCI was shown to be related to mortality (OR: 1.28, *P* = 0.002) [32]. Aydin et al. reported that mortality increased 3.6-fold in patients with high CCI scores and CCI was found a predictor of mortality [33].

In candidemia patients with COVID-19; higher procalcitonin, lactate, CRP, NLR, urea, and lower LCR were found in non-survivors than survivors. Additionally, higher urea, lactate, and procalcitonin levels were defined as independent predictors of mortality in candidemia with COVID-19, in this study. Cheng et al. concluded that acute kidney disease (elevated blood urea nitrogen) had a significantly higher risk for in-hospital mortality with COVID-19 [34]**.** Previously, acute kidney injury and the requirement for renal replacement therapy have been identified as independent risk factors for mortality of candidemia [1,35]. Considering these results; an assessment of other accompanying conditions (such as acute renal failure sepsis, and oxygenation) of COVID-19 may be important to reduce mortality for candidemia.

Corticosteroids are recommended in the treatment of critically ill patients who require oxygen or mechanical ventilation support [36]. In the present study, steroid administration was more common in candidemia patients with COVID-19 than non-COVID-19 controls. Additionally, the percentage of steroid administration was found to be significantly different between the COVID-19 groups (*P* = 0.01). However, there was no association found between steroid use and candidemia in COVID-19 patients (*P* = 0.695). Similarly, Omrani et al. reported that corticosteroids were not independently associated with candidemia in COVID-19 patients [14]. It was previously reported that candidemia with COVID-19 patients had more steroid administration than controls, but the authors did not evaluate the association between candidemia and steroid use [11,30]. Papadimitriou-Olivgeris et al. reported that corticosteroid administration in both pandemic and pre-pandemic periods was not associated with increased candidemia incidence. They mentioned that the increase in candidemia cannot be solely attributed to immunosuppression (corticosteroids, tocilizumab) of severe COVID-19 patients, but also to the increased workload of medical and nursing staff [9]. A multicenter study also did not demonstrate corticosteroids as risk factors for candidemia among patients with COVID-19 [37]. Although corticosteroids were thought to be associated with candidemia in studies before the pandemic, some studies suggested that there was no association [1,5,6,38]. According to the resuts of this study, It can be said that the presence of invasive procedures (such as CVC and MV) and the use of TPN were more effective in predicting candidemia in COVID-19 compared to corticosteroids.

In conclusion, characteristics and risk factors for candidemia are investigated among patients in ICUs with COVID-19 in this study. TPN, fever, and hypotension were identified as independent risk factors for candidemia in COVID-19 patients. The recognition of risk factors for candidemia may determine and prevent mortality in COVID-19. Also, early detection of warning signs for sepsis, such as fever and hypotension can predict candidemia in COVID-19 patients.

This study presents some limitations. The first was that data extracted from patient files may be incomplete because retrospective design of the study. Second, we were able to sample a portion of the entire candidemia population because the study included ICUs located in only two of our hospital's six buildings (general and oncology buildings). The other limitation, laboratory parameters were performed only at the time of admission. If parameters are measured at multiple time points (at candidemia onset etc.), the strength of results to predict the risk factors and mortality for candidemia may increase. However, this study had some strengths. In addition to various specific risk factors for the development of candidemia, we also evaluated mortality in patients with candidemia. Another strength was that we compared the COVID-19 groups with candidemia without COVID-19.

## Data availability statement

Data will be made available from the corresponding author (SK) on request.

## CRediT authorship contribution statement

**Sumeyye Kazancioglu:** Writing – review & editing, Writing – original draft, Project administration, Methodology, Investigation, Data curation, Conceptualization. **Hurrem Bodur:** Writing – review & editing, Writing – original draft, Visualization, Supervision, Methodology, Investigation, Conceptualization. **Ipek Mumcuoglu:** Writing – review & editing, Writing – original draft, Data curation, Conceptualization. **Aliye Bastug:** Writing – review & editing, Writing – original draft, Supervision, Investigation, Conceptualization. **Bahadir Orkun Ozbay:** Writing – review & editing, Writing – original draft, Software, Methodology, Investigation, Formal analysis, Data curation. **Omer Aydos:** Writing – review & editing, Writing – original draft, Software, Investigation, Formal analysis, Data curation, Conceptualization. **Bedia Dinc:** Writing – review & editing, Writing – original draft, Methodology.

## Declaration of competing interest

The authors declare that they have no known competing financial interests or personal relationships that could have appeared to influence the work reported in this paper.
